# Reforming pharmacy education in Japan: insights from the 2022 model core curriculum in a global context

**DOI:** 10.1080/20523211.2025.2609040

**Published:** 2026-01-14

**Authors:** Kayoko Takeda Mamiya

**Affiliations:** Department of Pharmaceutical Education, Faculty of Pharmaceutical Sciences, Hokkaido University of Science, Sapporo, Hokkaido, 006-8585, Japan

**Keywords:** Japanese pharmacy education, needs, model core curriculum, global pharmacy education

## Abstract

In response to demographic changes, technological innovation, and the increasing complexity of healthcare, Japan revised its Model Core Curriculum (MCC) for pharmacy education in 2022 (hereafter, the 2022 MCC). This reform aims to develop pharmacists with lifelong competencies, the ability to integrate emerging science and technology into practice, and strong interprofessional collaboration competencies. It represents a shift from traditional knowledge-based education to competency- and outcome-based education that addresses evolving societal needs in healthcare and pharmacy. This commentary outlines the key principles and evolution of the 2022 MCC. Faced with challenges such as population aging, declining birth rates, and healthcare system modernisation, Japanese pharmacy education is undergoing significant change. The 2022 MCC enhances practical clinical training, promotes community-oriented healthcare, and encourages scientific inquiry and problem-solving competencies among pharmacists. To support international collaboration and knowledge exchange, an English translation of the 2022 MCC Revised Edition has been published. As many countries face similar demographic and technological challenges, Japan’s experience may provide useful insights to support the modernisation of pharmacy education globally.

## 1. Introduction

In today’s rapidly changing healthcare landscape, professionals – particularly those in regulated fields such as pharmacy – must not only possess specialised knowledge but also be able to apply it effectively to meet diverse healthcare needs. This includes optimising pharmacotherapy for individual patients and promoting the overall health of local populations. To this end, it is essential to understand both the ongoing advancement of science and technology and the evolving needs of society. Educational strategies designed to address these dynamic requirements are referred to as needs-based education (Anderson et al., 2009; International Pharmaceutical Federation, 2020a, 2020b). This approach emphasises the development of professionals capable of adapting to current and future healthcare challenges.

This report examines the evolution of Pharmacy education in Japan, focusing particularly on the 2022 revision of the Model Core Curriculum (MCC), which – aside from 2018 and 2021 reports has not been thoroughly addressed in prior literature (Ohtani, 2021; Ozawa, 2018). Finally, this comment presents a comparison of pharmacy education in Japan and other countries, along with a discussion of key observations and implications.

## 2. The transitions in Japanese pharmacy education since 2002

**Figure 1.**
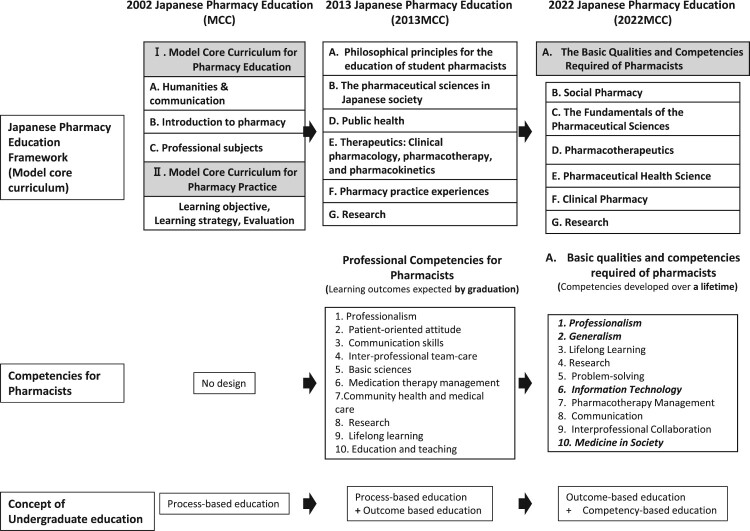
Transition in the Framework of Japanese Pharmacy Education since 2002. MCC: Model Core Curriculum; 2013MCC: Model Core Curriculum (2013 version); 2022MCC: Model Core Curriculum (2022 version).

The transitions in Japanese pharmacy education are illustrated in Figure 1. In 2002, the Japanese pharmacy education system was restructured from a four-year to a six-year program (hereinafter referred to as the 4-YP and the 6-YP, respectively), with the new system implemented in 2006 (Japan Society for Pharmaceutical Education, 2016; Ozawa, 2018). Notably, until 2002, pharmacy education in Japan lacked a structured foundation. That year marked the first establishment of a clear educational framework, which was based on the principles of process-based education. The 6-YP became the main track for training pharmacists, whereas the 4-YP was retained as a path for developing pharmaceutical scientists.

Following the introduction of the MCC for the 6-YP, several issues emerged chief among them was the difficulty universities faced in incorporating their own distinctive curricula due to the extensive volume of MCC. In response, the MCC was revised in 2013 (2013MCC), leading to an approximately 25% reduction in content volume (Japan Society for Pharmaceutical Education, 2016; Ministry of Education, Culture, Sports, Science and Technology, 2013; Ozawa, 2018). In the same year, the required competencies for pharmacists, defined as the expected learning outcomes at graduation, were formally established. The revised 2013MCC contained both process-based and outcome-based educational concepts.

In recent years, the scope of information in the healthcare domain has expanded considerably, fuelled by the emergence of big data and artificial intelligence (AI), alongside a growing emphasis on public health at the community level. To address these developments, the curricula for medicine, dentistry, and pharmacy were once again revised and partially standardised in 2022 (Ministry of Education, Culture, Sports, Science and Technology, 2022; Takeda & Arakawa, 2022). The updated framework, referred to as the 2022 MCC (Ministry of Education, Culture, Sports, Science and Technology, 2022; Takeda & Arakawa, 2022), contains both outcome-based and competency-based educational concepts. The 2022 MCC of competencies reflects a broader and more forward-looking perspective, placing greater emphasis on fostering lifelong development of professional competencies (Ministry of Education, Culture, Sports, Science and Technology, 2022; Takeda & Arakawa, 2022).

## 3. 2022MCC for Japanese pharmacy education revision overview

The MCC for Japanese pharmacy education is designed to enhance practical clinical skills through training in clinical settings, to acquire the knowledge and skills in the fundamentals of the pharmaceutical sciences, pharmacotherapeutics, public health, pharmacy practice experiences, etc., which are studied before graduation in the six-year pharmacy education system, in order to study and acquire the basic qualities and competencies required as pharmacists throughout life. The curriculum is designed to help students acquire the competencies to play an active role in society as pharmacists (Ministry of Education, Culture, Sports, Science and Technology, 2022).

**Figure 2.**
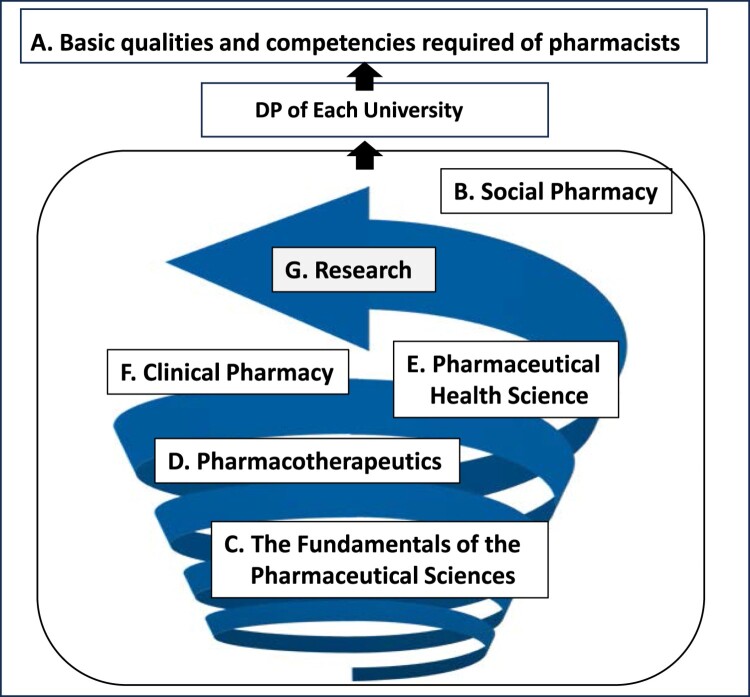
Schematic Structure of Japanese Pharmacy Education.

Pharmacists are expected to fulfil their duties in the manufacturing, dispensing and supply of medicines, to provide the public with appropriately quality-controlled medicines efficiently and without excess or deficiency, and to take on the social responsibility of widely contributing to pharmaceutical hygiene and the health promotion of patients and consumers. For these reasons, pharmacists must have sufficient qualities and competencies as healthcare professionals who are sincerely close to patients and consumers and proactively contribute to the promotion of community health, not only in health and healthcare, but also in nursing care and welfare. They must also acquire and utilise specialist knowledge and skills so that they can make appropriate scientific judgments and have the attitude to contribute to the development of medicine and pharmacy with a spirit of scientific inquiry. Therefore, A. (Basic Qualities and Competencies Required of Pharmacists, 10 competencies / clusters) explicitly outlines the essential qualities required of pharmacists, while B to G (B. Social Pharmacy, C. Fundamentals of the Pharmaceutical Sciences, D. Pharmacotherapeutics, E. Pharmaceutical Health Science, F. Clinical Pharmacy, G. Research) systematically present the key components necessary to develop these qualities over the course of a six-year educational program (Ministry of Education, Culture, Sports, Science and Technology, 2022) (Figure 2).

## 4. Basic policy for revisions

In this section, we describe the six core principles that guided the 2022 MCC (Ministry of Education, Culture, Sports, Science and Technology, 2022). These changes reflect a growing emphasis on developing pharmacists who can serve broader roles in healthcare delivery and community well-being, not merely as technical experts.

### 4.1. Educational content designed for pharmacists who can play an active role in a society undergoing major changes

In recent years, we have faced various problems, such as demographic changes, and these changes in social structure are expected to further intensify over time. In addition, as inter- and intra-healthcare teams progress as part of a comprehensive community care system, there is a greater need to improve the efficiency of objective tasks and enhance interpersonal tasks, and the roles and tasks of pharmacists in community healthcare, including during large-scale disasters, are undergoing significant changes. The content was designed to develop pharmacists who can provide safe, high-quality medical care as healthcare professionals and contribute to the improvement and promotion of public health in such a transforming society.

### 4.2. Development of a new 2022MCC that presents the basic qualities and competencies required of pharmacists to be aimed at throughout life

In the 2013 revision, there was a mix of a structure of academic outcome-based education with the basic qualities required as pharmacists at graduation and a process-based education that presented the general instructional objectives (GIOs) and specific behavioural objectives (SBOs).^1^ This was changed to a new development of learning outcome-based education with the basic qualities and competencies required as pharmacists as a lifelong goal.

### 4.3. Greater freedom for each university to responsibly implement the curriculum

In the 2013MCC, the subjects to be studied were described in detail as SBOs, and each university spent time covering them that it did not have time to incorporate university-specific content into its curriculum. The detailed SBOs were abolished, and the content to be studied was made into a ‘core,' allowing universities more freedom in curriculum development to enable responsible education on the basis of their respective philosophy and diploma policy (DP).

The 2022MCC has been revised to enable students not only to memorise many concrete facts but also to think about their common features and differences and acquire comprehensive academic skills that can be utilised to solve new challenges and problems. Each university aimed to develop its curriculum on the basis of its academic objectives.

### 4.4. The establishment of an educational system of clinical pharmacy

The emphasis is not on practical training in clinical settings (e.g. training for new early career training) with the aim of being able to practice as a specialist at individual facilities but rather on what to do as pharmacists for the benefit of the public in the future, what issues to find solutions for, and how to contribute to society. In this 2022MCC, an educational system called Clinical Pharmacy was established to produce pharmacists who can prevent diseases and provide responsible pharmacotherapy suited to individual patient conditions from the first year of university through cooperation between the university and medical fields.

### 4.5. Perspectives on human resource development for scientific exploration

To meet social needs, the 2022MCC aims to develop human resources that can scientifically explore, make discoveries, and find solutions to problems to contribute to the further development of healthcare.

### 4.6. Partial commonality with the 2022MCC for medical and dental education

From the perspective of promoting interprofessional healthcare teams, the content that should be common was discussed and harmonised on the occasion of the revision of the 2022MCC for education in medicine, dentistry, and pharmacy.

## 5. Structure of the revised 2022MCC for pharmacy education

In this section, we describe the structure of the 2022MCC for pharmacy education (Ministry of Education, Culture, Sports, Science and Technology, 2022) (Figure 2).

### 5.1. The text of 2022MCC for pharmacy education consists of the following main sections

A. Basic Qualities and Competencies Required by Pharmacists, B. Social Pharmacy, C. Fundamentals of the Pharmaceutical Sciences, D. Pharmacotherapeutics, E. Pharmaceutical Health Science, F. Clinical Pharmacy, G. Research. The structure of the 2022MCC is systematically designed to foster the development of quality ‘A' by enabling students to acquire major items C and D, build on major item B, and ultimately demonstrate major items E, F, and G as performance outcomes.

### 5.2. A. Basic qualities and competencies required of pharmacists

#### 5.2.1. Professionalism

Pharmacists are to have a rich sense of humanity and a deep awareness of the dignity of life, a sense of mission and responsibility to contribute to the maintenance and promotion of human health as pharmacists, a sense of ethics to respect the rights of patients and consumers and protect their interests, and make their best efforts to prevent the occurrence of health problems/harm (drug-related harm, medical accidents/errors, serious side effects, etc.) caused by pharmaceuticals and other products, realise medical care, welfare and public health that prioritises life and living with an altruistic attitude.

#### 5.2.2. Generalism

Achieve high quality medical, welfare, and public healthcare by understanding the physical, psychological, and social backgrounds of patients and consumers, and by taking a holistic and integrated view of the situation.

#### 5.2.3. Lifelong learning

As pharmacists responsible for medical, welfare, and public health care, set their own goals to achieve and continue to learn throughout their lives, while studying and teaching themselves and others.

#### 5.2.4. Research

From a pharmaceutical perspective, accurately identify issues in medicine, welfare, and public health, and contribute to the development of pharmacy by appropriately planning and practicing academic and research activities while acquiring scientific thinking for problems solution.

#### 5.2.5. Problem-solving

Acquire pharmacological knowledge and skills to understand the relationship of pharmaceuticals and other chemical substances to life and the environment from a professional perspective, to make appropriate scientific judgements, and to use these for diverse and advanced medical, welfare, and public health purposes.

#### 5.2.6. Information technology

Take an interest in advanced and cutting-edge technologies in society and actively utilise technologies related to epidemiology, artificial intelligence and big data, making use of their expertise as pharmacists and in compliance with ethics, laws, institutions, and norms related to information, science, and technology.

#### 5.2.7. Pharmacotherapy management

Independently plan, implement, and evaluate pharmacotherapy healthcare management including the appropriate supply of medicines, dispensing of medicines according to the situation, providing medication guidance/counselling and proposing patient-centred care to prescribers.

#### 5.2.8. Communication

Communicate empathetically and well with patients, consumers, and healthcare professionals to support their decision-making through the appropriate and smooth exchange of information.

#### 5.2.9. Interprofessional collaboration

Understand the roles of all the professionals who make up an inter-healthcare team partnership and practice quality patient- and consumer-centred healthcare, welfare, and public health, while building equal relationships with each other.

#### 5.2.10. Medicine in society

Take on roles in medical care, welfare, and public health from a broad perspective that spans from the local and international perspectives, from pre-disease and prevention to treatment, prognosis management, and end-of-life care.

Each university should not directly adopt the A. Basic Qualities and Competencies Required of Pharmacists described in the 2022MCC as its DP but should define it in accordance with its own educational policy. In other words, with reference to the content of A. Basic Qualities and Competencies Required by Pharmacists, a DP that can be evaluated at the time of graduation is formulated from the academic objectives listed in major items B to G, taking into full account the uniqueness of each university, human and material resources, the educational environment, and an effective Curriculum Policy and Admission Policy for the six years of study (Figure 2).

In the future, it is hoped that further seamless education from pregraduate education to postgraduate training, etc., will foster a lifelong attitude toward studying with A. Basic Qualities Required by Pharmacists as a lifelong goal.

## 6. International trends in pharmacy education

**Figure 3.**
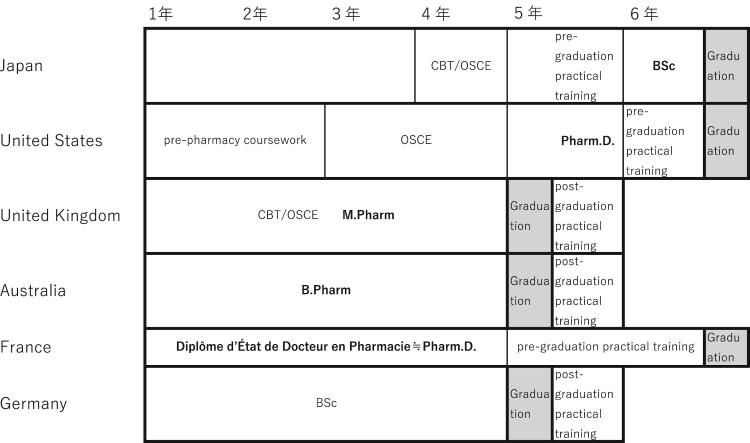
International Comparison of Pharmacy Education Structures. CBT: computer-based testing; OSCE: Objective Structured Clinical Examination; BSc: Bachelor of Science; M. Pharm.: Master of Pharmacy; B. Pharm: Bachelor of Pharmacy; Pharm.D.: Doctor of Pharmacy.

The duration, structure, and system of pharmacy education vary across countries. These differences are summarised in Table 1. Figure 3 is based on data compiled by Matsuoka (2018) and Kirino (2022), who investigated pharmacy education systems in various countries. Table 1 presents the clusters or domains of learning outcomes or competencies for pharmacy students or pharmacists in each country, as identified by the authors from English-language sources. The greater number and broader scope of competencies defined in Japan, compared to the United Kingdom and the United States, may be attributed to the fact that pharmacist licenses in Japan are permanent and do not require renewal (General Pharmaceutical Council, 2018.; International Pharmaceutical Federation, 2020b; Ministry of Health, Labour and Welfare, 1960), and that there is no pharmacy technician system in place (Accreditation Council for Pharmacy Education (n.d.); General Pharmaceutical Council, 2017). In contrast, in many other countries, advanced competencies are defined and used as the basis for license renewal systems.

**Table 1.** Changes in competencies and learning outcomes for pharmacy students and pharmacists by country: 2010s vs. 2020s

	Target	2010s	2020s
		Cluster/domain of learning outcome or competency	Cluster/domain of learning outcome or competency
Japan	Undergraduate student	2013 (Ministry of Education, Culture, Sports, Science and Technology, 2013)	2022 (Ministry of Education, Culture, Sports, Science and Technology, 2022)
		1. Professionalism	1.Professionalism
		2. Patient-oriented attitude	2. Generalism
		3. Communication skills	3. Lifelong learning
		4. Inter-professional team-care	4. Research
		5. Basic sciences	5. Problem-solving
		6. Medication therapy management	6. *Information technology*
		7. Community health and medical care	7. Pharmacotherapy management
		8. Research	8. Communication
		9. Lifelong learning	9. Interprofessional collaboration
		10. Education and teaching	10. *Medicine in society*
United States	Undergraduate student	2015 (Accreditation Council for Pharmacy Education, 2015)	2025 (Accreditation Council for Pharmacy Education, 2025)
		1.Foundational knowledge	The ACPE standards were revised in 2025; however, the learning outcomes /competency framework remain the same as those established in 2015
		2.Essentials for practice and care	
		3.Approach to practice and care	
		4.Personal and professional development	
United Kingdom	Undergraduate student/trainee pharmacist (initial education and training of pharmacists)	2011 (General Pharmaceutical Council, 2011)	2021 (General Pharmaceutical Council, 2021)
		1.Expectations of a pharmacy professional	*1. Person-centred care and collaboration*
		2.The skills required in practice	2. Professional practice
			*3. Leadership and management*
			*4. Education and research*
Australia	2010 for Pharmacist 2020 for Undergraduate student	2010 (Pharmacy Board of Australia, 2010)	2020 (Australian Pharmacy Council, 2020)
		1. Provide primary health care	1. Deliver person-centred care
		2. Communicate effectively	2. Communicate with other health professionals to support patient care
		3. Behave professionally	3. Exhibit professional behaviour and integrity
		4. Maintain and develop professional competence	4. Maintain and develop professional competence
		5. Contribute to public and preventive health	5. Promote and contribute to public health and wellbeing
		6. Manage and supply medicines	6. Manage pharmacy services and medicines supply chains
			*7. Apply evidence-based practice in clinical decision-making*
			*8. Use digital and health informatics tools to improve outcomes*
			*9. Demonstrate cultural safety and responsiveness*
A Global Competency Framework Provided by FIP	Early career pharmacists	2012 (International Pharmaceutical Federation, 2012)	2020 (International Pharmaceutical Federation, 2020a)
		1. Pharmaceutical public health competencies	1. Pharmaceutical public health (*+Emergency response*)
		2. Pharmaceutical care competencies	2. Pharmaceutical care
		3. Organisation and management competencies	3. Organisation and management
		4. Professional/personal competencies	4. Professional/personal (*+ digital literacy, inter-professional collaboration, leadership and self-regulation*)

In Japan, the United States, and France, practical training is incorporated into undergraduate pharmacy education. In contrast, countries such as the United Kingdom, Australia, and Germany do not include practical training as part of their undergraduate programs (Matsuoka, 2018; see Figure 3). Moreover, in the United Kingdom, the General Pharmaceutical Council (GPhC) specifies the outcomes expected of pharmacy students and trainee pharmacists in its Standards for the Initial Education and Training of Pharmacists (GPhC, 2011, 2021). Similarly, the International Pharmaceutical Federation (FIP) has proposed a Global Competency Framework targeting early-career pharmacists (FIP, 2012, 2020a), culminating in a validated and internationally relevant 2020 version (Bajis et al., 2023). Bajis et al. (2023) report that this competency framework is effective and applicable across diverse national contexts, as it was developed with international relevance in mind. These observations suggest that, despite differences in educational structures, the core competencies and achievement goals required of pharmacy students at graduation or early-career pharmacists are largely consistent across countries (Table 1).

One notable trend from the 2010s to the 2020s is the increasing inclusion of competencies or learning outcomes related to information technology, digital health, and digital literacy, reflecting the evolving role of pharmacists in response to changing societal needs. Moreover, no countries have reduced the number or scope of competencies or learning outcomes during this period. These developments indicate that the roles and expectations of pharmacists are expanding globally, even in the era of AI. Therefore, pharmacy professionals are expected to take on greater responsibility in addressing these emerging societal needs.

## 7. Conclusions

In recent years, scientific and technological advancements have been remarkable, and the practices and workflows of pharmacists are expected to undergo significant changes. Furthermore, Pharmacists are expected to play an increasingly important role in disease prevention, which is especially vital in Japan for maintaining its universal healthcare system. Therefore, those involved in Pharmacy education – regardless of country – bear the responsibility of fostering professionals who can meet contemporary societal needs by accurately understanding and responding to ongoing social changes. Sharing Japan’s revised 2022 MCC internationally may provide a valuable reference for countries aiming to align their pharmacy education systems with societal needs – particularly those facing similar demographic and technological pressures or those that have not yet revised their curricula in response to the demands of the 2020s.

## Data Availability

Data supporting the findings of this study are available in References ‘Ministry of Education, Culture, Sports, Science and Technology (2022) and Model Core Curriculum for Pharmacy Education (https://www.mext.go.jp/content/20250206-mxt_igaku-100000058_01.pdf)’.

## Footnotes

1 GIOs and SBOs: In the 2013 MCC, the general instructional objectives (GIOs) (outcomes that students obtain through their studies) were set to acquire basic qualities, and the SBOs, specific behavioural objectives (individual practical competences that students should acquire to reach the GIO), were set.

## Abbreviations

2013MCC: model core curriculum (2013 version); 2022MCC: model core curriculum (2022 version); AI: artificial intelligence; DP: diploma policy; FIP: international pharmaceutical federation; GIOs: general instructional objectives; GPhC: General Pharmaceutical Council; MCC: model core curriculum; SBOs: specific behavioural objectives.
